# Transcriptional Response of the Mussel *Mytilus galloprovincialis* (Lam.) following Exposure to Heat Stress and Copper

**DOI:** 10.1371/journal.pone.0066802

**Published:** 2013-06-25

**Authors:** Alessandro Negri, Catherina Oliveri, Susanna Sforzini, Flavio Mignione, Aldo Viarengo, Mohamed Banni

**Affiliations:** 1 Department of Environmental and Life Sciences, Università del Piemonte Orientale Vercelli Novara Alessandria, Alessandria, Italy; 2 Laboratory of Biochemistry and Environmental Toxicology, ISA, Chott-Mariem, Sousse, Tunisia; Glasgow Caledonian University, United Kingdom

## Abstract

Global warming is a major factor that may affect biological organization, especially in marine ecosystems and in coastal areas that are particularly subject to anthropogenic pollution. We evaluated the effects of simultaneous changes in temperature and copper concentrations on lysosomal membrane stability (N-acetyl-hexosaminidase activity) and malondialdehyde accumulation (MDA) in the gill of the blue mussel *Mytilus galloprovincialis* (Lam.). Temperature and copper exerted additive effects on lysosomal membrane stability, exacerbating the toxic effects of metal cations present in non-physiological concentrations. Mussel lysosomal membrane stability is known to be positively related to scope for growth, indicating possible effects of increasing temperature on mussel populations in metal-polluted areas. To clarify the molecular response to environmental stressors, we used a cDNA microarray with 1,673 sequences to measure the relative transcript abundances in the gills of mussels exposed to copper (40 µg/L) and a temperature gradient (16°C, 20°C, and 24°C). In animals exposed only to heat stress, hierarchical clustering of the microarray data revealed three main clusters, which were largely dominated by down-regulation of translation-related differentially expressed genes, drastic up-regulation of protein folding related genes, and genes involved in chitin metabolism. The response of mussels exposed to copper at 24°C was characterized by an opposite pattern of the genes involved in translation, most of which were up-regulated, as well as the down-regulation of genes encoding heat shock proteins and “microtubule-based movement” proteins. Our data provide novel information on the transcriptomic modulations in mussels facing temperature increases and high copper concentrations; these data highlight the risk of marine life exposed to toxic chemicals in the presence of temperature increases due to climate change.

## Introduction

In recent decades, the increase in CO_2_ levels and other greenhouse gases has been recognized as a major environmental problem at the global level (December 1997 – Kyoto). One consequence of this increase is climate change; continuous temperature increases may represent an important risk to the evolution of ecosystems [1]. Recent investigations have studied and modeled the possible effects of climate change on the marine environment [2], [3], [4], [5]. Most of these studies were carried out at the organismal level and are intended to clarify the effects of temperature increase on the physiology of marine organisms [6]; [7] as well as the growth and reproduction of the population and community.

At the cellular and molecular levels, studies have evaluated the roles of genes encoding proteins known to be involved in the stress response. It is well known that toxic chemicals, as well as environmental parameters such as temperature, may affect the levels of heat shock proteins, which contribute to the restoration of the structure of partially denatured proteins as well as in protein folding and translocation. Changes in proteins involved in the responses to oxidative stress, pollutants, and temperature have been recently investigated as well [8]. However, only a few investigations have attempted to clarify the response to temperature by aquatic organisms exposed to toxic chemicals [9]; [10]; [7], a scenario that may reflect risk to the marine environment in general and to coastal areas in particular.

Copper is an essential metal that becomes toxic when present in excessive amounts in the ecosystem. A European Union program, Marine Ecosystem Evolution in a Changing Environment, determined that copper is released into the marine environment in large, and is present at high concentrations along the coast as well as in the water column in the open sea, its concentration is often particularly high in sediments and interstitial waters [11]; [12]; [13].

Mollusks, particularly *Mytilus sp.*, are well studied at the molecular, cellular, tissue, and organismal levels. These mollusks are usually used in large monitoring programs to study the accumulation of pollutants in their tissues and the consequent effects of this accumulation on biological processes [14]; [15]; [16]. The use of biomarkers, considered as the changes that may occur, from the molecular to the organism level, is often employed to evaluate the physiological status of organisms exposed to environmental stressors [15]. For example, the blue mussel (*Mytilus galloprovincialis* Lam.) is known to experience toxicity when exposed to copper at various temperatures (16–24°C) [7].

The current investigation had two main aims. Firstly, we sought to identify the effects of simultaneous changes in temperature and copper concentrations on lysosomal membrane stability (LMS), a digestive gland-sensitive biomarker, in *M. galloprovincialis* [17]. Several studies indicated that changes in LMS are positively related to changes in scope for growth, a parameter predictive of effects at the organism-population level [18]. We also investigated the Cu loads in digestive gland and gills as well as MDA accumulation in gills tissues. Secondly, we used a DNA miroarray with ∼1673 genes to analyze the transcriptomic response of the mussels to temperature and various sublethal concentrations of copper. The transcriptional response was studied in the gills, where metals accumulate at high levels; the microarray data were confirmed by quantitative reverse-transcription PCR (qRT-PCR).

## Materials and Methods

### Animals and treatments

Specimens of *M. galloprovincialis* (Lam.), 5–6 cm shell length, were purchased from an aquaculture mussel farm in Arborea (Sardinia, Italy) in November 2010. These specimens were transferred to aquaria at a density of 1 animal/L in clean, aerated seawater collected offshore. Experiments were carried out at three temperatures (16°C, 20°C, and 24°C). After an acclimation of 6 days, a period of time sufficient to stabilize at control temperature the mussel physiological response [19], groups of mussels were kept in 20 polypropylene plastic vessels (four replicates per treatment) and underwent semi-static exposure to 2.5 µg/L, 5 µg/L, 10 µg/L, 20 µg/L, or 40 µg/L copper for 4 days. One set of animals was maintained in seawater with no addition of copper at the three experimental temperatures. Seawater of the desired temperature was renewed every day, and copper was added together with a commercial algal preparation (30 mg animal^−1^ day^−1^) (Liquifry; Interpret Ltd., Dorking, Surrey, UK). Only female individuals (scored by microscopic inspection of gonad biopsies) were selected for subsequent analysis to avoid gender-based bias in gene expression. After exposure to heat and copper, digestive glands and gills were rapidly removed, frozen in liquid N2, and stored at −80°C for MDA and chemical analysis. A second set of tissues was kept at −20°C in an RNA-preserving solution (RNA Later; Sigma-Aldrich) for transcriptome analysis, and a third set was mounted on aluminum chucks and frozen in super-cooled N-hexane as previously described [17] for histochemical determination of LMS.

### Chemical analysis

Copper levels were determined in mussel digestive gland and gills (∼0.5 g of a 1∶1 homogenate in double-distilled water) by inductively coupled plasma-mass spectrometry (VG Plasma Quad 3, VG Elemental). Samples were added to 5 mL of concentrated 65% nitric acid and placed in a microwave oven for mineralization. The samples were then filtered on a nitrocellulose membrane (0.45 µm). Procedure validation was performed using the Std CRM 145 R reference material containing known amounts of metal.

### MDA determination

Gill tissues (0.5–1 g) were homogenized in 2 volumes of buffer containing Tris HCl 20 mM (pH 7.4) and 0.1% mercapto-ethanol. The homogenate was centrifuged at 18,000×g (4°C) for 20 min. MDA concentration was determined in the supernatant as described by [20].

### LMS assay

LMS was evaluated in cryostat sections (10 µm) of five digestive glands obtained with a “Leica cryostat” apparatus at −27°C, as described by Moore [17]. Staining intensity of lysosomes was determined by studying the slide at 400×magnification with an inverted Axiovert microscope (Zeiss), connected to an Axiocam digital camera (Zeiss). Digital image analysis was carried out using the Scion Image software package (Scion Corp. Inc.) from 8-bit grayscale images.

### Microarray hybridization and analysis

Competitive dual-color microarray hybridization was performed with the Mytarray V1.1 platform [21]; fluorescence-labeled cDNA probes were obtained by direct labeling in the presence of modified Cy3- and Cy5-dCTP (Perkin Elmer). The procedure was carried out as described by [19] using 0.5 µg of an anchored oligodT(19)VN. Total RNA was extracted from female individual gills pieces using acid phenol-chloroform precipitation according to [22], with TRI-Reagent (Sigma-Aldrich). RNA was further purified by precipitation in the presence of 1.5 M LiCl2, and the quality of each RNA preparation was confirmed by UV spectroscopy and TBE agarose gel electrophoresis in the presence of formamide, as described in [23]. Laser scanning of microarrays was performed with an Agilent G2565CA scanner (Agilent Technologies, Inc., USA) at 5-µm resolution. Sixteen-bit TIFF images were analyzed with Genepix 6.0 (Axon) to extract raw fluorescence data from each spot.

The experimental design accounted for two complete “triangular loops” in which each RNA sample from the tissue of mussels exposed to temperature T was hybridized with that exposed to temperatureT+4°C (temperatures were equal to 16°C, 20°C, and 24°C). One design accounted only for temperature variations, while the other included copper-exposed animals (40 µg/L) at 16°C, 20°C, and 24°C. Direct comparisons were performed between RNA samples obtained from copper-exposed and unexposed animals at 16°C, 20°C, and 24°C to complete the triangular loop and to cover all conditions. Each experimental condition had at least four biological replicates of RNA samples from single individual female animals using the day-swap procedure, for a total of 36 experiments.

### Statistical analysis

Computational and statistical analysis of microarray data was performed using the Linear Mode for Microarray Analysis (LIMMA) software [24]. Offset background subtraction, loess normalization, and least-squares regression were employed, along with moderated t-tests and empirical Bayes statistics. Gene expression was considered to be significantly different in the test condition versus the reference condition when the log-odd value (B) was higher than 0. The analysis procedure was carried out essentially as described in [19]. Microarray data were clustered with the Genesis software [25]; [26].

MIAMI-compliant microarray data, including a detailed description of the experimental design and each hybridization experiment, were deposited in the Gene Expression Omnibus with identifier “GSE41899”. The following link provides access to the deposited data http://www.ncbi.nlm.nih.gov/projects/geo/query/acc.cgi?acc=GSE41899.

### Functional genomics analysis

Functional characterization of mussel genes represented on the microarray was based on Gene Ontology (GO) annotation and was carried out with Blast2GO [27] using default parameters. Briefly, 1,673 mussel sequences with EMBL IDs were subjected to the annotation analysis; 880 sequences had no BLASTX hits [28], while another 63 sequences did not map to GO terms. Putative annotation for 873 mussel sequences was established based on GO terms for the first 20 BLASTX hits or based on protein domains obtained from Inter Pro Scan [29]; [30]. GO term enrichment was evaluated with hypergeometric statistics (p<0.05); the distribution of GO terms in each set of interest was compared against the set reflecting the entire microarray sequence catalogue.

### qRT-PCR

qRT-PCR was carried out with the same RNA extract used for microarray hybridization. Relative mRNA abundances of the mussel genes encoding metallothionein (mt10, EMBL ID AJ625847; mt20, not present on the array) and the gene encoding calreticulin (AJ624756) were evaluated with SYBR Green I chemistry (EvaGreen®dye; Bio-Rad Laboratories; [30]). The mRNA abundances of the genes encoding eukaryotic translation elongation factor 1 alpha 1 (AJ624922) and chitinase (AJ624093, AJ625569, and AJ624637) were evaluated in multiplex Taqman assays according to [30]. For the gene encoding fk506-binding protein (AJ624969) and ribosomal protein genesribo-s12 (AJ626437), ribo27-s27 (AJ625324), and ribo-s19 (AJ625447), multiplex Taqman assays were set up ex novo. Probes and primer pairs (Table S1) were designed using Beacon Designer v3.0 (Premier Biosoft International, Inc.). All primers and dual-labeled Taqman probes were synthesized by MWG-Biotech Gmbh (Germany).

cDNA (25 ng RNA reverse-transcribed to cDNA) was amplified in a CFX384 Real-Time PCR detection system (Bio-Rad Laboratories) with iQTM Multiplex Power mix (Bio-Rad Laboratories) according to the manufacturer's instructions for the triplex protocol. All multiplex combinations accounted for the following dual fluorescence tags: 6-carboxyfluorescein/Black Hole (BH) 1, 6-carboxy-2′,4,4′,5′,7,7′-hexachlorofluorescein/BH1, and Texas Red/BH2. Briefly, cDNA was amplified in the presence of 1X iQTM Multiplex Power mix, 0.3 µM each primer, and 0.1 µM each probe (Table S1) in a final volume of 10 µL. Relative expression data were geometrically normalized to18S rRNA (L33452),an invariant actin isotype (AJ625116; [30]), and ribosomal proteinriboL27(AJ625928), which were selected from a list of genes whose expression did not vary over more than 50 conditions (including toxic treatments, stages of the life cycle, and various tissues).

A specific duplex Taqman assay was developed to amplify 0.25 ng of RNA reverse-transcribed to cDNA in the presence of 0.1 µM of each dual-labeled probe (hexachlorofluorescein/BH1 for actin and Texas Red/BH2 for 18S rRNA) and 0.1 µM and 0.4 µM of forward and reverse primer, respectively, for 18S rRNA and actin (Table S1). For all Taqman assays, the thermal protocol was as follows: 30 s at 95°C, followed by 40 cycles of 10 s at 95°C and 20 s at 60°C. qRT-PCR was performed with four biological replicates and three technical replicates. For the mt-10, mt-20, and calreticulin assays, the thermal protocol was as presented by [31]. Statistical analyses were carried out on the group mean values using a random reallocation test [32].

### Statistical analysis

The results for LMS, Cu accumulation and MDA measurement are presented as the mean ±SD of 10 samples. The Statistica Software, version 6.0, computer software package (Statsoft. Inc. 2002) was used for statistical analysis. The normality of the distribution was tested using the Shapiro-Wilk test. To assess multiple comparisons, a parametric one-way analysis of variance (ANOVA) was performed on data, with a Tukey's test.

## Results

**Figure 1:**
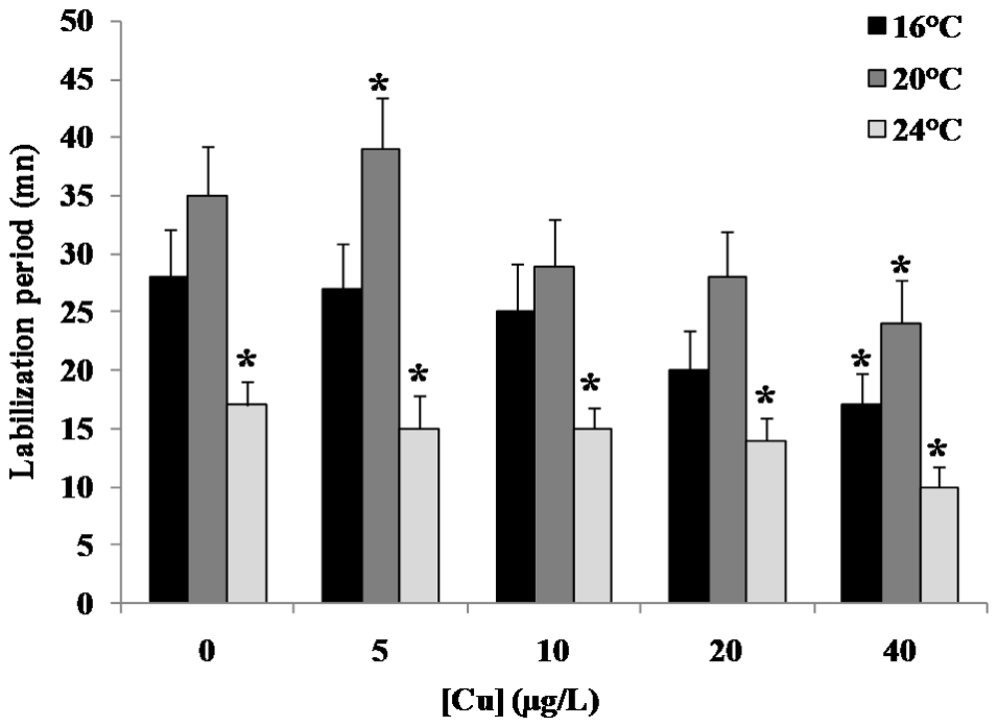
Effect of exposure to copper along with a temperature gradient on lysosomal membrane stability in*Mytilus galloprovincialis* digestive gland. Mussels were exposed for 4 days to Cu (0; 5; 10; 20 and 40 µg/L) along with a temperature gradient (16°C; 20°C and 24°C). Data, expressed as labilisation period (n = 10), were analyzed by ANOVA+ Tukey's post test. *: Statistically significant differences (P<0.01) in comparison with control condition (16°C without Cu supply).

**Figure 2:**
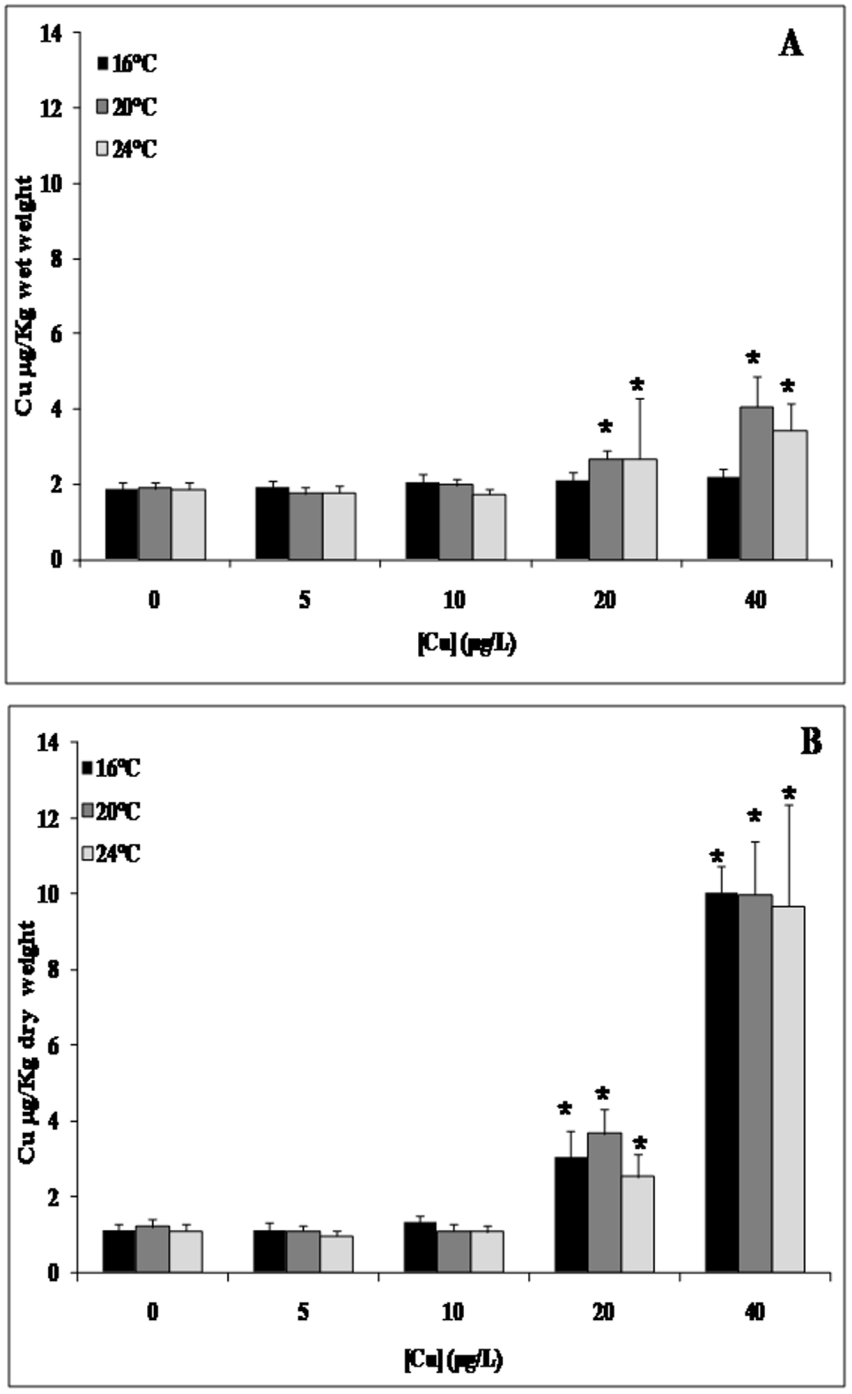
Copper accumulation in*Mytilus galloprovincialis* digestive gland (Panel A) and gills (Panel B) in animals exposed for 4 days to increasing Cu concentrations (0; 5; 10; 20 and 40 µg/L) along with a temperature gradient (16°C; 20°C and 24°C) Data, expressed in µg/g dry weight (n = 10), were analyzed by ANOVA+ Tukey's post test. *: Statistically significant differences (P<0.01) in comparison with control condition (16°C without Cu supply).

**Figure 3:**
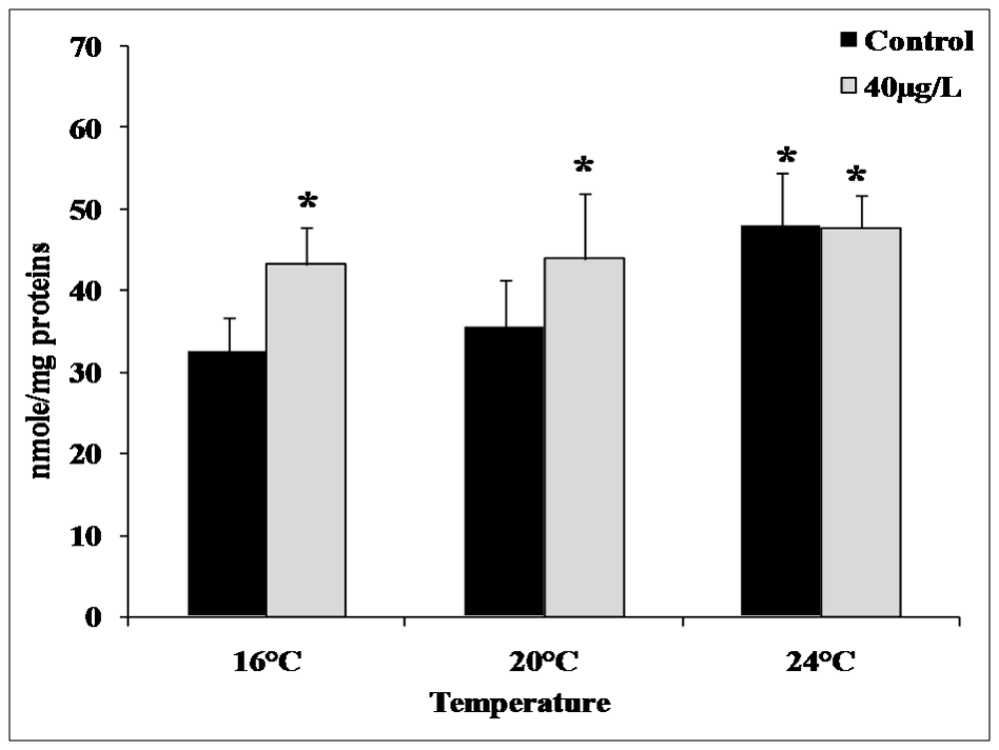
Malondialdehyde (MDA) accumulation in*Mytilus galloprovincialis* gills (Panel B) in animals exposed for 4 days to 40 µg/L Cu along with a temperature gradient (16°C and 24°C). Data, expressed in nmole/mg proteins (n = 10), were analyzed by ANOVA+ Tukey's post test. *: Statistically significant differences (P<0.01) in comparison with control condition (16°C without Cu supply).

The effects of copper exposure and temperature gradient on LMS in the mussel digestive gland appear in Fig. 1. We detected a pronounced dose-dependent response at 24°C and 40 µg/L copper. Copper concentrations were higher in the gills than in the digestive glands mainly under the highest Cu exposure concentration (Fig. 2). Moreover, copper bioaccumulation in mussel tissues exhibited a dose-dependent trend that was essentially independent of temperature. MDA accumulation significantly increased in the gills after exposure to 40 µg/L copper at 16°C and 24°C/Cu and 24°C with a pronounced increase at 24°C when compared to 16°C (Fig. 3).

**Figure 4:**
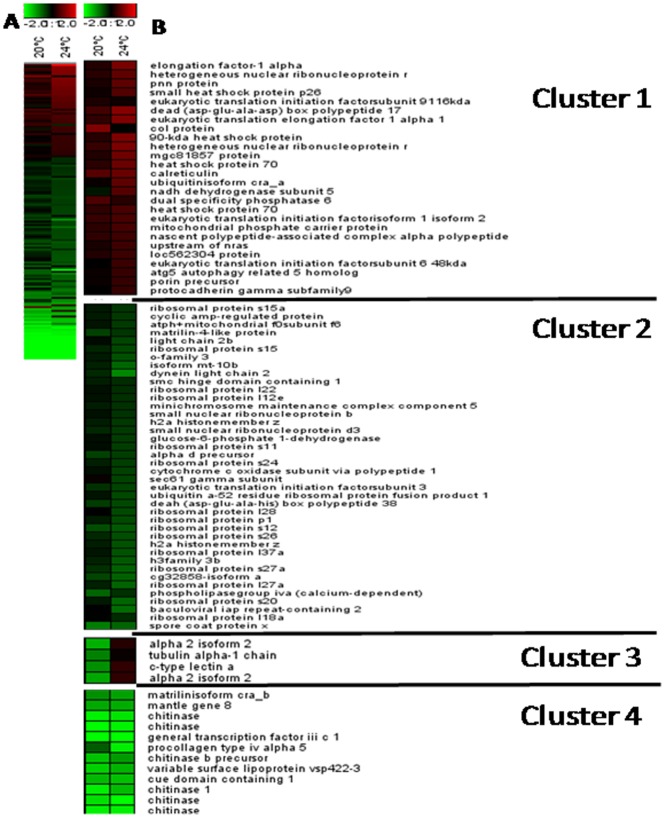
*Mytilus galloprovincialis* gene expression profiles of digestive gland tissue along with the temperature gradient. The heat map (A) (Pearson correlation, complete linkage algorithm) and the decomposition of gene expression profile (B) report the log2 relative expression level with respect to the selected reference condition (16°C). 161 differentially expressed genes were generated in at least one condition. Microarray data were analyzed using the Linear Mode for Microarray Analysis (LIMMA) software as described in [23]. B statistics with adjusted p value, 0.05 and B.0 were used as threshold for rejection of the null hypothesis (no variation). Supporting information to Figure 2 is present in Table S2 and Table S3. The k-means algorithm was used for the computation of different gene expression trends in the set of 161 unique genes whose expression was modulated in female gills along with the temperature gradient (table S3). K-means is an iterative procedure aimed to reduce the variance to a minimum within each cluster [24]; [25].

Large-scale transcriptional profiling was performed to identify the main molecular mechanisms involved in the response of mussels to copper exposure and heat stress. Using a 1.673-feature cDNA microarray, we generated transcriptome profiles for female gills exposed to 20°C and 24°C for 4 days, and we compared these data to profiles from control animals maintained at 16°C. Microarray analysis revealed distinct patterns for 161differentially expressed genes (DEGs; differential expression under at least one condition (Fig. 4A; Tables S2, S3). Of the 161 DEGs, only 39 genes were shared between the 20°C and 24°C datasets (Table 1).

**Table 1:** Number of DEGs in mussels*Mytilus galloprovincialis* exposed to heat stress.

**Condition**	**20°C**	**24°C**	**Shared**	**Total**
**DEGs**	64	136	39	161
**Up-regulated**	9 (14%)	44(32.5%)	0	53 (23%)
**Down-regulated**	55 (86%)	92 67.5%)	39 (100%)	108 (67%)

To obtain more insight into the major patterns of gene expression following exposure to increased temperature, we performed K-means clustering (Fig. 4B; Table S4). Furthermore, we identified significantly enriched GO terms to reveal the biological processes contributing to the responses to exposure to 20°C and 24°C (Table 2; Table S5) and common to the responses to both temperatures (Table 2). Analysis of the 64 DEGs in animals exposed to 20°C (versus 16°C) highlighted the following contributing biological processes: protein polymerization, cellular protein metabolic processes, microtubule-based movement, and cellular biosynthetic processes (Table S4). In addition, the 136 DEGs obtained from animals exposed to 24°C were significantly associated with 10 biological processes, largely composed of “translation” processes (23 DEGs). Response to unfolded proteins was represented by five up-regulated DEGs in animals exposed to 24°C (table S4). Four heat shock proteins were included in this class (heat shock protein 70, AJ624049 and AJ624615; heat shock protein 90, AJ625915; small heat shock protein 26, AJ624926; and calreticulin, AJ624756);only the gene encoding calreticulin was differentially expressed in animals exposed to 20°C.

**Table 2:** GO term over-representation analysis of DEGs in the gills tissue of mussels exposed to heat stress.

Condition	Level	Go Term	N	(up)	Gene ID
**16°C–20°C**	4	Regulation of cellular protein metabolic process	3	3	AJ625912, AJ516752, AJ625915
	4	Microtubule-based movement	3	0	AJ625595, AJ625032, AJ625866
	4	Cellular biosynthetic process	3		AJ624768, AJ516582, AJ623925
	3	Protein polymerization	3	0	AJ625595, AJ625032, AJ625866
**16°C–24°C**	6	Translation	23	5	AJ626184, AJ625269, AJ516392, AJ624922, AJ625132, AJ624829, AJ624086, AJ625934, AJ625678, AJ625006, AJ625356, AJ625324, AJ625383, AJ625549, AJ625548, AJ624757, AJ626296, AJ625376, AJ624844, AJ516412, AJ625505, AJ624454, AJ623665
	4	RNA processing	5	1	AJ516537, AJ624991, AJ516404, AJ625133, AJ626226
	3	Larval development	6	1	AJ516886, AJ516404, AJ625236, AJ624922, AJ626296, AJ516600
	3	Chromosome organization	5	0	AJ516582, AJ516441, AJ624454, AJ516600, AJ516663
	3	Cellular component assembly	6	1	AJ516886, AJ624768, AJ516582, AJ623925, AJ516600, AJ516663
	3	Response to unfolded protein	5	5	AJ624615, AJ625915, AJ624926, AJ624756AJ624049
	3	Ribosome biogenesis	6	0	AJ516392, AJ625133, AJ625356, AJ625383, AJ625376, AJ623665
	2	Growth	5	1	AJ516886, AJ516404, AJ625236, AJ624922, AJ516600
	4	Regulation of cellular biosynthetic process	7	3	AJ516537, AJ625915, AJ626184, AJ625269, AJ625132, AJ516412, AJ624454
	3	System development	5	1	AJ516886, AJ516404, AJ625236, AJ624922, AJ626296
**In Common**	3	Chitin catabolic process	6	0	AJ624087, AJ625569, AJ625051, AJ624093, AJ624637, AJ625778

Gene Ontology terms enrichment analysis was carried out comparing the GO term frequency distribution into each condition against that in the whole microarray set (hypergeometric statistics, p,0.05). Only the lowest node per branch of the hierarchical structure of the Gene Ontology that fulfills the filter condition - cut off 3 sequences- was reported. Showed are: experimental condition; Level, level in the GO tee of biological processes; GO Term, over-represented feature; N, number of mussel sequences associated to each GO term; up, Number of up-regulated genes; Gene ID, EMBL accession number of each sequence found. the over-represented GO terms in heat stresses animals versus 16°C (hypergeometric stats, p,0.05).

We also carried out qRT-PCR to confirm and refine the relative expression levels of 11 homologue genes belonging to the most important K-means clusters, including metallothionein (mt10, EMBL ID AJ625847; mt20, not present on the array), calreticulin (AJ624756), eukaryotic translation elongation factor 1 alpha 1 (AJ624922), three chitinase variants (AJ624093, AJ625569, and AJ624637), fk506-binding protein (AJ624969) and ribosomal protein genes ribo-s12 (AJ626437), ribo27-s27 (AJ625324), and ribo-s19 (AJ625447). Microarray and qPCR data showed a positive relationship in all cases (Fig. S1).

RNA was extracted from the gills of animals exposed to copper and various temperatures. Dual-color microarray hybridizations revealed 119 DEGs (56% down-regulated), 66 DEGs (54.5% down-regulated), and 177 DEGs (45.1% down-regulated) for animals exposed to 16°C, 20°C, and 24°C, respectively, between copper-exposed animals and controls (Table S3). GO analysis of the 119 DEGs in animals exposed to copper at 16°C highlighted nine biological processes, including the up-regulation of 16 genes involved in translation (Table 3; Table S6). We detected the up-regulation of genes involved in ribosome biogenesis, macromolecular complex assembly, post-embryonic development, and organ development and growth. For the 66 DEGs at 20°C, we uncovered four GO-enriched biological processes, including up-regulation of genes involved in “protein folding” and “RNA processing” and down-regulation of genes belonging to the class “response to stimulus” (Table 3; Table S6). The transcriptional response of mussels exposed to copper and a temperature of 24°C was completely different from the response of animals exposed only to 24°C, and also from those exposed to copper and a temperature of 20°C. The main pattern was characterized by the up-regulation of 38/41 genes contributing to the GO process of “translation,” 12 genes involved in “ribosome biogenesis,” and 6/10 genes implicated in “cellular macromolecular complex assembly,” as well as down-regulation of 7 genes associated with “microtubule-based movement” (Table 3; Table S6).

**Table 3:** GO term over-representation analysis of DEGs in the gills tissue of mussels exposed to copper along with heat stress.

Condition	Level	Go Term	N	(up)	Gene ID
**16_Cu/16°C**	6	Translation	16	14	AJ625495, AJ625269, AJ626374, AJ516491, AJ624125, AJ625132, AJ626437, AJ625006, AJ625324, AJ625356, AJ625548, AJ625546, AJ623547, AJ624301, AJ625604, AJ624649
	4	Organ development	7	3	AJ516886, AJ625490, AJ625655, AJ516404, AJ624125, AJ625488, AJ626467
	3	Ribosome biogenesis	7	4	AJ516491, AJ625133, AJ626437, AJ625356, AJ623352, AJ623547, AJ624649
	2	Growth	5	4	AJ516886, AJ516404, AJ626179, AJ626329, AJ623342
	4	Post-embryonic development	5	4	AJ516886, AJ516404, AJ625488, AJ626179, AJ626329
	4	Nervous system development	5	3	AJ516886, AJ625655, AJ516404, AJ624125, AJ625488
	4	Chitin catabolic process	5	0	AJ624093, AJ624637, AJ624087, AJ625051, AJ625569
	3	Cellular macromolecular complex assembly	5	4	AJ626179, AJ626329, AJ625083, AJ516796, AJ516663
	3	Nuclear mRNA splicing, via spliceosome	5	5	AJ516537, AJ516404, AJ626179, AJ626329, AJ625083
**20_Cu/20°C**	4	Response to stimulus	9	6	AJ624926, AJ625244, AJ625131, AJ623342, AJ625488, AJ625311, AJ624260, AJ624898, AJ625490
	4	RNA processing	5	4	AJ624597, AJ624828, AJ626179, AJ623352, AJ626329
	3	Protein folding	5	3	AJ624926, AJ625244, AJ624969, AJ623698, AJ624898
	4	Multicellular organsmal development	5	2	AJ624597, AJ625441, AJ625488, AJ626179, AJ625655
**24_Cu/24°C**	6	Translation	41	38	AJ624649, AJ623547, AJ625549, AJ625548, AJ625546, AJ625447, AJ625934, AJ624248, AJ625342, AJ624732, AJ625244, AJ624925, AJ624829, AJ626437, AJ516491, AJ626296, AJ624429, AJ624426, AJ516392, AJ624488, AJ624324, AJ626091, AJ625324, AJ624871, AJ624125, AJ625874, AJ516873, AJ626184, AJ625376, AJ623665, AJ516412, AJ624503, AJ625957, AJ624109, AJ625366, AJ625006, AJ516364, AJ516361, AJ624454, AJ516752, AJ624844
	3	Ribosome biogenesis	12	12	AJ624649, AJ623547, AJ625342, AJ626437, AJ625133, AJ516491, AJ516392, AJ516873, AJ625376, AJ623665, AJ625366, AJ516361
	3	Cellular macromolecular complex assembly	10	6	AJ516796, AJ625595, AJ624686, AJ625091, AJ626329, AJ516582, AJ625866, AJ626179, AJ516600, AJ516663
	3	Microtubule-based movement	7	0	AJ623937, AJ516796, AJ625595, AJ516886, AJ625091, AJ625473, AJ625866
	3	DNA conformation change	7	7	AJ626296, AJ516886, AJ626329, AJ625027, AJ626179, AJ516600, AJ516404

Gene Ontology terms enrichment analysis was carried out comparing the GO term frequency distribution into each condition against that in the whole microarray set (hypergeometric statistics, p,0.05). Only the lowest node per branch of the hierarchical structure of the Gene Ontology that fulfills the filter condition - cut off 5 sequences- was reported. Showed are: experimental condition; Level, level in the GO tee of biological processes; GO Term, over-represented feature; N, number of mussel sequences associated to each GO term; N up, Number of up-regulated genes; Gene ID, EMBL accession number of each sequence found. The over-represented GO terms in copper exposed animals versus relative control (16°C, 20°C and 24°C) (hypergeometric stats, p,0.05).

Co-exposure to copper and elevated temperatures resulted in a new transcriptional profile for the genes encoding heat shock proteins versus exposure to heat only (Table 4). In animals exposed to 20°C plus copper, we detected the up-regulation of the genes encoding 6 heat shock proteins: heat shock protein 70(AJ624049, AJ624615), heat shock protein 90(AJ625915), small heat shock proteins 26(AJ624926) and 27 (AJ625244), and the fk506-binding protein (AJ624969). Interestingly, up-regulation of only the genes encoding small heat shock proteins 26 and 27 was maintained in animals exposed to 24°C plus copper (table 4).

**Table 4:** Log 2-fold change (M values) of the 7 heat shock proteins differentially expressed in gills of mussel exposed to copper along with the temperature gradient.

Gene ID	Gene Name	Experimental condition				
		20°C	24°C	16°C/Cu	20°C/Cu	24°C/Cu
**AJ624049**	Heat shock protein 70	-	0,57	-	0,83	-
**AJ624926**	Small heat shock protein p26	-	0,64	-	0,63	0,78
**AJ624756**	Calreticulin	0,67	0,82	-	-	-
**AJ624615**	Heat shock protein 70	-	0,83	-	0,83	-
**AJ625915**	90-kda heat shock protein	-	0,97	-	1,19	-
**AJ624969**	Fk506-binding protein	-	-	0,56	0,67	0,62
**AJ625244**	Heat shock 27 kda protein 1	-	-	-	0,93	2,00

**Figure 5:**
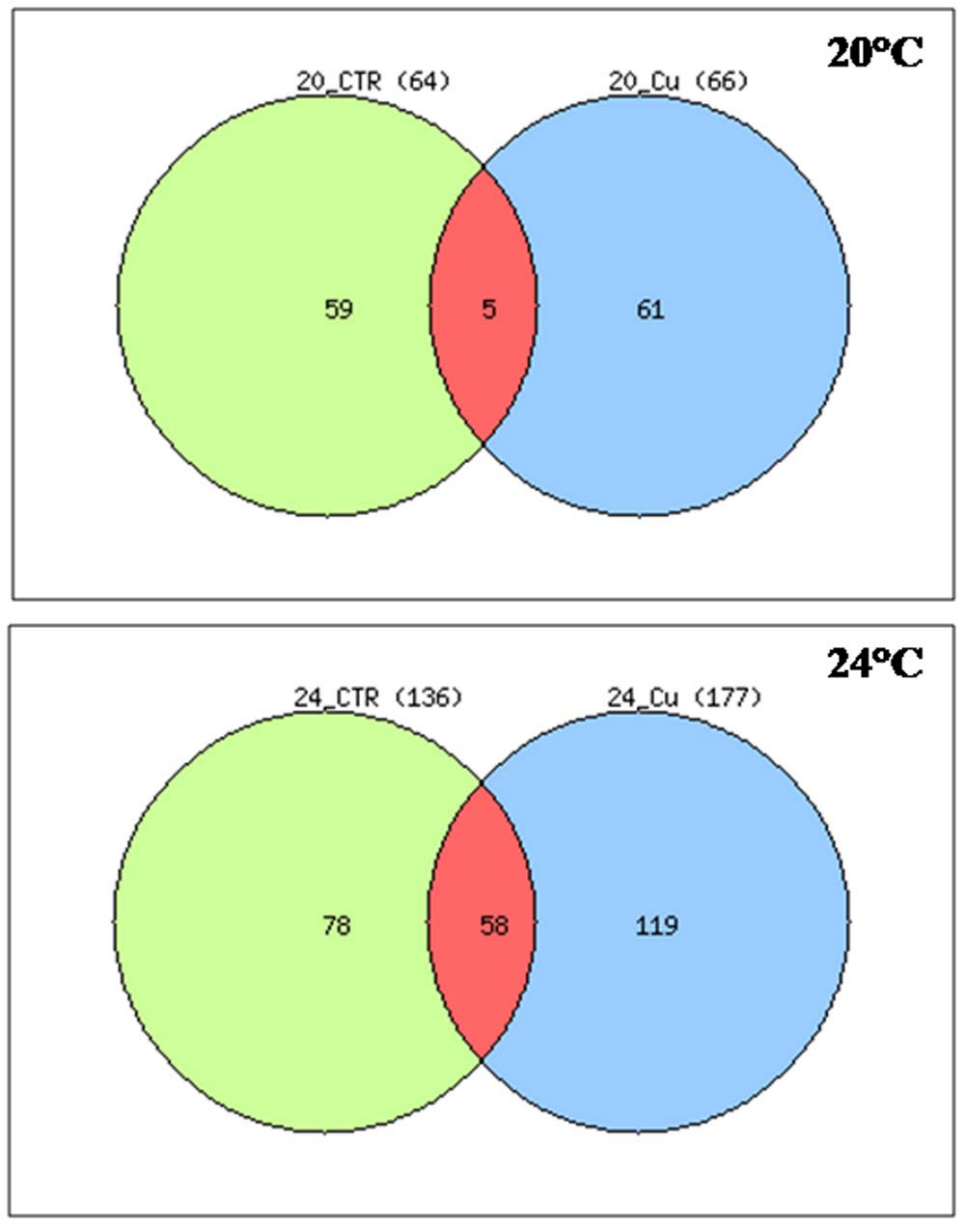
Venn diagram representation of gene expression patterns. The diagram clearly depicted that only 5 DEGs shared between 20°C and 20°C plus Cu and 58 DEGs between 24°C and 24°C plus Cu. All DEGs are obtained respect to the control condition 16°C.Data used to generate the Venn-diagram were obtained from microarray analysis (Table S3).

We performed a qualitative comparison of the DEGs resulting from temperature exposure alone and the DEGs identified following co-exposure to heat and copper. A Venn diagram was generated for each temperature (Fig. 5), and GO term enrichment was assessed to identify qualitative differences between biological processes shared between the strict temperature effect and the co-effect of copper and temperature (Tables 2 and 3).

## Discussion

An increasing body of evidence indicates that global climate change will exert important effects on living organisms, including aquatic organisms such as mussels that inhabit tidal zones. Several recent investigations sought to understand the biological strategies employed by mussels confronting climate change and invading new suitable areas [33]; [34]; [6]. However, tidal zones are subject to severe contamination episodes, such as heavy metal hot spots [14].

Here we report the acute effects of increasing temperature and copper exposure on the biological response of the mussel *M. galloprovincialis*. LMS analysis revealed a toxic effect following exposure to increased copper concentrations, an effect that became pronounced at higher temperatures (Fig. 1). LMS has been reported to be a very sensitive cellular marker for assessing environmental impact [15]; [18]; [35]; [36]. Previous studies described clear correlations between LMS and other high-level ecotoxicological endpoints, such as immune response and cell death [37]; [38]. The direct relationship between LMS and scope for growth allows LMS to be linked to potential effects at the organismal and population levels, as proposed by Allen and Moor [36]. These findings of a negative effect of copper exposure in combination with a temperature gradient motivated our investigation of the adaptive/toxicity molecular mechanisms of mussels to heat stress and copper exposure.

We performed chemical analysis of copper uptake in a variety of mussel tissues to determine the most suitable tissue for revealing information about the mussel response to copper exposure and heat stress. As expected and as it was previously described by Baines and Fisher [39], we observed a higher accumulation of copper in gills than in the digestive gland, an organ with higher metabolic function [40]; [41]. These data may be related to the exposure route; in many experiments, copper was added to the seawater, which may yield higher accumulation measurements for the tested xenobiotics, particularly in gills in cases of acute exposure [42]. Therefore, we selected gills for our transcriptional analysis of mussels exposed to copper and a temperature gradient. Gills were also used to evaluate the transcriptomic [33] and proteomic [6] responses of mussels to heat stress and other environmental stresses [43].

The ability of a stressed organism to adjust its cellular processes via transcriptional control can allow it to cope with the alteration of cellular functions and to avoid cellular damage that could also lead to death. Our microarray and GO analyses identified 64 DEGs at 20°C and 136 DEGs at 24°C and revealed that distinct biological processes were involved in the adaptation to higher temperatures. The response of mussels exposed to 24°C is characterized by the up-regulation of protein folding (five genes) and the down-regulation of translation-related genes (23 genes). Among proteins represented in our microarray and involved with protein folding, calreticulin and HSP27 seem to play pivotal roles in the response to heat stress. Calreticulin is known to bind misfolded proteins, preventing their export from the endoplasmic reticulum to the Golgi apparatus [44]. Up-regulation of calreticulin and HSP27 was recently reported to be highly up-regulated in *M. galloprovincialis* with respect to *M. trossulus* exposed to heat stress, thus explaining the invasive success of *M. galloprovincialis* in the southern and central coasts of California [33].

We detected marked down-regulation at 24°C of 23 DEGs related to the translation process, although this effect is difficult to interpret because these genes encode proteins that are implicated in multiple cellular pathways. Of these 23 down-regulated DEGs, 13 encode ribosomal protein subunits (table S5); their down-regulation may indicate that mRNA-directed protein synthesis is reduced in mussels at 24°C. Temperature is known to interfere with protein synthesis, as the abundances of proteins involved in protein synthesis, protein degradation, ATP supply, and structural proteins change in response to heat exposure in sturgeon larvae [10]. Ribosome biogenesis is temperature-dependent in bacteria [45] and Connolly and Hall [46] suggested that both transcription and translation are regulated in zebrafish in response to environmental stresses such as high temperature.

Copper exposure and heat stress are potent inducers of oxidative stress [47]; [48]; [33]; [6]. Accordingly, the MDA accumulation observed here (Fig. 3) suggests marked oxidative stress damage in mussels exposed to copper and increasing temperatures. However, our transcriptional data do not indicate modulation of oxidative stress-related genes in heat-stressed animals. This observation maybe due to the limit of the microarray used in this study, which only contained 1,673 sequences, increasing the probability of failing to detect some transcriptional changes involved in the oxidative-stress response. Specifically our microarray lacked probes for the genes encoding superoxide dismutase, catalase, and glutathione S-transferase, which are typically involved in the response to oxidative stress.

The transcriptional response of mussels exposed to 20°C was marked by the down-regulation of the genes encoding two tubulin isoforms and actin, which are involved in microtubule-based movements. Cytoskeletal protection was previously proposed as a potential mechanism for increased thermo-tolerance in mussels [33]. In contrast to the reported proteomic response of warm-adapted *M. galloprovincialis*, in which tubulin levels were generally higher at 24°C and 28°C than at 32°C [6], in the present work the gene encoding tubulin was down-regulated at 20°C and was unchanged at 24°C relative to 16°C, probably because the experimental conditions (exposure period and route) were not identical to the previous study.

Functional analysis of the 39 DEGs common to the 20°C and 24°Cdatasets indicated the down-regulation of the genes encoding six chitinase variants at both temperatures, suggesting the involvement of chitin metabolism in the response to increased temperature. The pattern of three chitinase variants (AJ624093, AJ625569, and AJ624637) was confirmed by mean of qRT-PCR (figure S1). In chitin-containing organisms, chitinases are essential for maintaining normal life-cycle functions such as morphogenesis [49], cell division, and immunity [50]. In mussels and other marine invertebrates, chitinases play a role in digestion and in the control of growth and remodeling processes; the activities of the chitinases have been reported to be highly correlated with food availability [31]. In this study, sustained down-regulation of these genes in copper- and/or heat-stressed mussels maybe a specific, toxic effect of higher temperatures.

The toxic effects of copper on numerous aquatic organisms have been studied intensely over the past 20 years [51]. To our knowledge, this is the first report of the transcriptional changes in mussels exposed to copper along a temperature gradient. Our data indicate a differential modulation of mRNA abundance in mussels co-exposed to copper and 16°C, 20°C, and 24°C. Compared to the effect of temperature alone, the mussel's response to exposure to copper and 24°C was mainly characterized by a clear increase in the number of up-regulated genes involved in “translation” and the number of down-regulated genes linked with “microtubule-based movement”. We only detected the persistence of the up-regulation of the genes encoding HSP27/HSP26 and FK506-binding protein when HSP70/HSP90 and calreticulin were unpaired.

Heat shock was previously reported to reduce the translation of proteins other than molecular chaperones, in part to avoid exposing nascent polypeptide chains to denaturing conditions [52]. This protective strategy is confirmed by our data in mussels exposed to 24°C. Similar data were reported by Tomanek and Zuzow [6], who observed clear increases in the levels of the molecular chaperones HSP70 and HSP90 in *M. galloprovincialis* and *M. trossulus* exposed to acute heat stress. Moreover, Lockwood *et al* [33] reported that small heat shock proteins are responsive to heat stress in *M. galloprovincialis*.

In this study we report a distinct transcriptional profile of molecular chaperones in mussels exposed to 24°Cand copper when compared with mussels exposed to 24°C alone, in which only small heat shock proteins were up-regulated. Conversely, 38 genes involved in translation were up-regulated in mussels exposed to 24°C plus copper versus 24°C exposure alone. These data suggest a negative interaction between copper and higher temperatures on the adaptive response of mussels to heat stress, and thus may underscore an ecological risk due to temperature increases in heavy metal-contaminated seawaters.

This study is the first report of the involvement of ribosomal transcripts in the response to copper exposure, probably due to an increase in the ribosome synthesis rate to support cellular activities such as protein translation, transcriptional activation, and mRNA stabilization during sublethal copper exposure. This transcriptional up-regulation was found to be very important in the presence of higher temperatures. Our data suggest that mussels adopt a compensatory strategy in response to the effects of exposure to sublethal copper levels, a strategy that is mainly characterized by up-regulation of genes involved in protein synthesis.

Small heat shock proteins such as HSP27, HSP26, and FK506-binding protein are likely to act as chaperones for maintaining cytoskeletal structural elements during stress [53]. Moreover, the cytoskeleton is likely to be critical for thermo-tolerance in mussel tissues, particularly in gills [33]. However, [33] carried out their observations at 32°C, while our observations were performed at 24°C in the presence of copper. Our data show that heat stress (24°C) and copper exposure in mussels lead to the up-regulation of genes involved in cytoskeletal protection.

## Conclusion

Global warming is an emerging threat to ecosystems worldwide. This work constitutes a first attempt to clarify how an increase in temperature of 5–8 degrees over the next 100–150 years, which is assumed by modelers, may exert negative effects on marine organisms, particularly those inhabiting coastal environments subject to anthropogenic pollutants.

The exposure of mussels to copper along a temperature gradient led to oxidative stress and resulted in transcriptional changes. Our results confirm the important contributions of heat shock proteins, including the folding and unfolding of misfolded proteins, in the response to heat stress. In mussels exposed to increased temperatures, we observed down-regulation of genes linked to translation, as well as an opposite expression pattern of these genes when mussels were exposed to increasing temperatures in the presence of copper.

## Supporting Information

Figure S1 **QPCR data of 10 selected targets obtained from the cluster analysis (Additional information to** **Table 2** **,** **table 3** **and** **Figure 4** **).** Gene expression was performed respect to 16°C and was normalized against Actin, 18S and Ribo L27. * Significantly different from reference condition (16°C), *p<005 threshold cycle random reallocation test according to [31], n = 4.
(PPTX)

Table S1 **Q-PCR primers and Taqman probes.** Given are: #, a progressive number; Gene ID, EMBL or NCBI gene Identifier; Taqman probe, sense primer and antisense primer sequences. All sequence are given 5′ to 3′. Legend: AJ625847, Mt-10 AJ624756, cartiticuline; AJ624922, eukaryotic translation elongation factor 1 alpha 1; AJ624093, AJ625569, and AJ624637; 3 chitinase variants; AJ624969, fk506-binding protein; AJ626437, ribosomal protein genes ribo-s12; AJ625324, ribosomal protein genes ribo27-s27a; AJ625447, ribosomal protein genes ribo-s19; AJ625928; ribosomal protein genes ribo-L27; AJ625116, actin; L33452, 18S ribosonal RNA.
(DOCX)

Table S2 **The total array sequence names, their description and their associated GOs(Blast2GO output) (Additional information to** **Figure 4** **).**
(XLS)

Table S3 **M-Values of the 161 DEGs in at least one condition during the exposure to 20°C and 24°C (16°C is considered as the reference) (Additional information to** **Figure 4B** **).**
(XLSX)

Table S4 **Details about the number, the unique ID and their M values in the two conditions (20°C and 24°C) associated to each cluster (Additional information to** **Fig. 4B** **).**
(XLSX)

Table S5 **Functional genomics analysis of Gills DEGs in animals exposed to 20°C and 24°C (against 16°C) (Additional information to** **table 3** **).**
(XLSX)

Table S6 **Functional genomics analysis of Gills DEGs in animals exposed to Cu along with the temperature gradient (Additional information to** **table 3** **).**
(XLSX)
