# Supplementary material for: Transcriptional Response of the Mussel Mytilus galloprovincialis (Lam.) following Exposure to Heat Stress and Copper
Source: PLoS One. 2013 Jun 25;8(6):e66802. doi: 10.1371/journal.pone.0066802 (PMC3692493; doi:10.1371/journal.pone.0066802)
Supplement: Figure S1 — QPCR data of 10 selected targets obtained from the cluster analysis (Additional information to Table 2 , table 3 and Figure 4 ). Gene expression was performed respect to 16°C and was normalized against Actin, 18S and Ribo L27. * Significantly different from reference condition (16°C), *p<005 threshold cycle random reallocation test according to [31], n = 4. (PPTX) [file pone.0066802.s001.pptx]

## Slide 1
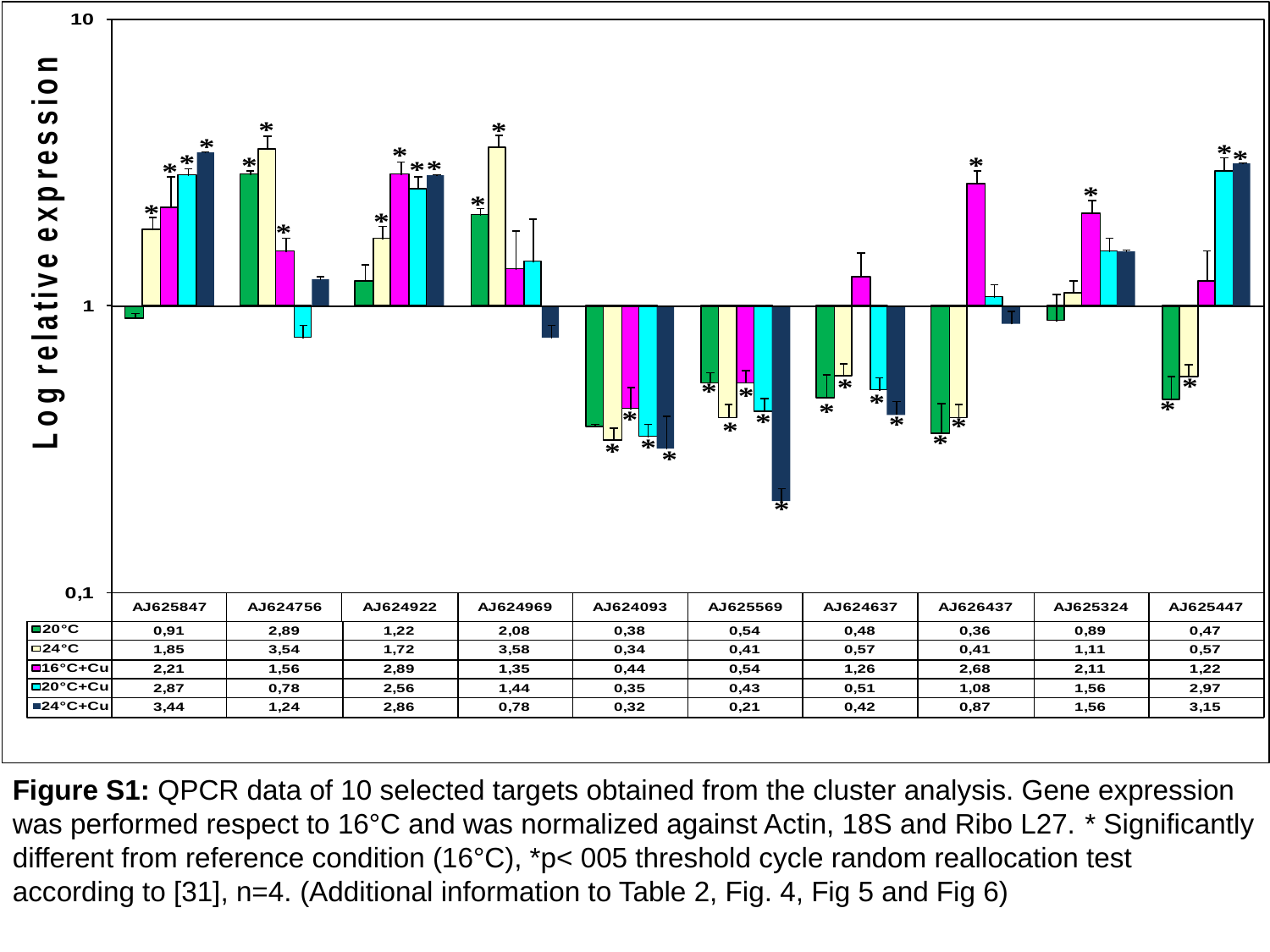

Figure S1: QPCR data of 10 selected targets obtained from the cluster analysis. Gene expression was performed respect to 16°C and was normalized against Actin, 18S and Ribo L27. * Significantly different from reference condition (16°C), *p< 005 threshold cycle random reallocation test according to [31], n=4. (Additional information to Table 2, Fig. 4, Fig 5 and Fig 6)
