# Supplementary material for: Transcriptional Response of the Mussel Mytilus galloprovincialis (Lam.) following Exposure to Heat Stress and Copper
Source: PLoS One. 2013 Jun 25;8(6):e66802. doi: 10.1371/journal.pone.0066802 (PMC3692493; doi:10.1371/journal.pone.0066802)
Supplement: Table S1 — Q-PCR primers and Taqman probes. Given are: #, a progressive number; Gene ID, EMBL or NCBI gene Identifier; Taqman probe, sense primer and antisense primer sequences. All sequence are given 5′ to 3′. Legend: AJ625847, Mt-10 AJ624756, cartiticuline; AJ624922, eukaryotic translation elongation factor 1 alpha 1; AJ624093, AJ625569, and AJ624637; 3 chitinase variants; AJ624969, fk506-binding protein; AJ626437, ribosomal protein genes ribo-s12; AJ625324, ribosomal protein genes ribo27-s27a; AJ625447, ribosomal protein genes ribo-s19; AJ625928; ribosomal protein genes ribo-L27; AJ625116, actin; L33452, 18S ribosonal RNA. (DOCX) [file pone.0066802.s002.docx]

Table S1. Q-PCR primers and Taqman probes

| Gene _ID | Probe | Sense Primer | Antisense Primer |
| --- | --- | --- | --- |
| AJ625256 | AACTTTTCCACCACGCCCGCCATT | AACATATACAGGCAAAGCACTACA | TCACCATCCGTAATGATAATTGCA |
| AJ624593 | AGCTTGTCCTCCTGTCTGGCCTCG | TGATGCAGTTCAACAGGAAGGT | ATTGCCAATGATCAAACACACACT |
| AJ625894 | TCCACCTTCAAGGGCTGTGACTCTGCT | CGGGAGGAGATGGACAGTTCA | CGGCTTAGATCCTGTTGCTGAA |
| AJ625621 | AAGTCCTCGCTTCCTCAGTCTCTCAACA | CACAGGTGAATCCAAAGATGTTGT | ACTCATCAATGGGGTCTATCATGT |
| AJ625655 | CTCCATGTGCCCTCTGAGTGAAACTTGT | TCAGTGATGATCCTAGATTAGGCA | CGTTCCTCTCTTTCCATCTGTAAC |
| AJ624922 | ACAAACTGTCGCCGTAGGAGTCATCAAA | CGTTTTGCTGTCCGAGACATG | CCACGCCTCACATCATTTCTTG |
| AJ624702 | TCATCTTGGTAATAGCCTCCGCAAATGC | AATACAAGAGATGTTGCTGCTACA | TTTGAACTGTTCCCATTCTGACTT |
| AJ624502 | AGAGGACCAACACCAACAACAATGACTG | AACGAGAACTTTGAATTCCAGAGA | CTCAGTAATCGGGCATTTTCCATA |
| AJ625661 | TCGGTGTTCTGTTCTCTACCCTCAATGC | TGCTGAGCTACTTCAAGGATGAG | TTTGATGAAGACCTCCCTCCAATT |
| AJ623584 | ACCCTTGTCACAGTCTTCTTAACCCACG | CTTATTGCACCTATTCTTACCGGC | TTCGTCTGTAAACTGTCCAACAAA |
| AJ624361 | AGCATCAATGTCTGTTGGTTTACCATCCG | GCAGTTTACATATACAGGCAAAGC | TTGCTTCTTTTACACGTCTTTGTG |
| AJ516774 | TGAACCGTCAGGTTGTCCTGCTGACCA | ACACAGCGGCAAATGGGATT | TGCGTAGACTGCTAAGCATGTT |

All sequences are given in 5'-3' direction
